# Cortical changes associated with an anterior cruciate ligament injury may retrograde skilled kicking in football: preliminary EEG findings

**DOI:** 10.1038/s41598-025-86196-4

**Published:** 2025-01-16

**Authors:** Daghan Piskin, Gjergji Cobani, Tim Lehmann, Daniel Büchel, Jochen Baumeister

**Affiliations:** Department of Sport & Health, Exercise Science & Neuroscience Unit Universität Paderborn, Warburger Straße 100, 33098 Paderborn, Germany

**Keywords:** Anterior cruciate ligament injury, Sports injuries, Football, Movement variability, Electroencephalography, Neurophysiology, Neuromuscular junction, Sensorimotor processing, Orthopaedics, Motor control

## Abstract

Anterior cruciate ligament injuries (ACLi) impact football players substantially leading to performance declines and premature career endings. Emerging evidence suggests that ACLi should be viewed not merely as peripheral injuries but as complex conditions with neurophysiological aspects. The objective of the present study was to compare kicking performance and associated cortical activity between injured and healthy players. Ten reconstructed and 15 healthy players performed a kicking task. Kicking biomechanics were recorded using wearable inertial measurement unit sensors. Cortical activity was captured with a 64-electrode mobile electroencephalography. Multiscale entropy (MSE) analysis of biomechanics revealed increased variability in foot external rotation among injured players. Source-derived event-related spectral perturbations indicated significant differences in posterior alpha and frontal theta oscillations between the two groups. Furthermore, kick-related complexity of these regions as indexed by MSE was reduced in injured players at medium and coarse scales. Our findings suggest sensorimotor changes during kicking in injured players, which may necessitate compensatory strategies involving augmented attention at the cost of processing visuospatial information. This conflict may hinder the integration of task-relevant information across distributed networks. Our study provides preliminary insights into the neurophysiological implications of ACLi within football context and underscores the potential for prospective research.

## Introduction

The rupture of the anterior cruciate ligament (ACL) is one of the most common injuries in football, characterized by a high incidence rate among players^1–3^. The successful return-to-play following surgical reconstruction of the ACL (ACLR) is influenced by various factors, including the type of sport involved^4^. The consequences of ACL injuries can be particularly severe in football, as studies indicate significant reductions in player performance post-ACLR^5,6^. Following ACL injury, players often experience fewer minutes per game and season, participate in fewer games, complete fewer passes, and score fewer goals^7,8^. Furthermore, Szymski et al.^5^ highlight that injured football players tend to have shorter career lengths due to diminished levels of play.

Understanding the implications of ACL injuries in football necessitates the physiological exploration of sport-specific skills in injured players. Kicking is a fundamental movement in football, essential for advancing or passing the ball and scoring goals^9,10^. Besides physical coordination, executing a kick requires the integration of visuospatial and proprioceptive information into coordinated motor behavior, placing considerable demands on cortical activity^11,12^. Kicking with precision engages characteristic responses from the posterior and frontal brain regions involved in visuospatial and attentional processes, respectively^13,14^. These processes play a crucial role in developing neural strategies that enhance the likelihood of successful kicks on target^15,16^. However, ACL injuries may disrupt kicking accuracy by interfering with these processes. Research indicates that individuals with ACL injuries exhibit differences in brain activity compared to healthy controls during both simple motor and postural tasks^17–22^. To compensate for altered afferent input coming from the knee, injured individuals develop cortical strategies^23^. The higher activation and connectivity of posterior and frontal regions while performing a motor task introduce the exploitation of visual and attentional resources for proprioceptive compensation^17–21^. For kicking, this may imply an overlap of cortical resources used for task demands and sensory compensation. Prioritizing one over another may challenge kicking performance due to insufficient sensory compensation or constrained processing of task-related information^24^. The findings of Cordeiro et al.^25^ indicate increased biomechanical variability and present preliminary behavioral evidence suggesting changes in performance among injured players while kicking. Still, it remains unclear from a neurophysiological perspective if a potential interplay between task demands and compensatory strategies deteriorates kicking performance.

With its high temporal resolution and portability, electroencephalography (EEG) has emerged as a widely used mobile neurophysiological method for investigating movement-related cortical dynamics^26^. In the context of ACL research, studies utilizing EEG have the potential to uncover differences in brain activity between injured and healthy individuals while they perform simple, postural and dynamic tasks^17,18,21,27,28^. For brief movements such as kicking, time-locked analyses facilitate the examination of rapid cortical dynamics that occur before and during movement execution. Three recent studies employing event-related spectral perturbation (ERSP) analyses have provided important insights into the cortical modulations observed in successive phases of kicking^12,15,16^. Specifically, spectral perturbations observed in posterior alpha and frontal theta activity during kicking were associated with improved accuracy^15,16^. While linear metrics of EEG offer valuable information regarding temporal and regional activation related to task-specific cortical activity^12,29^, non-linear metrics such as multiscale entropy (MSE) assess the complexity of the EEG signals, serving as a proxy of the brain’s information processing capacity^30^. By analyzing complexity across various temporal scales using MSE, studies have demonstrated that pathologies or increased cortical load can constrain the amount of information processed within local and distributed neural networks^31–33^. The integration of linear and non-linear metrics can offer a more nuanced perspective on cortical changes associated with injury, ultimately enhancing the interpretation of EEG data with the novel employment of MSE in mobile settings^34^.

Based on this background, our study aims to compare kicking performance and associated cortical activity between healthy and injured football players who have undergone an ACLR. We will evaluate kicking performance in terms of accuracy and consistency by measuring trial-to-trial non-linear fluctuations in the short-distance passing motion through MSE^16,35^. Further, we will analyze cortical modulations in the posterior and frontal regions during kicking using ERSPs^12,15,16^. In order to investigate the impact of an ACL injury on the brain complexity related to kicking, we will also conduct MSE analysis on the activity of these two regions^30^. On the behavioral level, we hypothesize that the results will show reduced accuracy and higher complexity for kicking biomechanics in the ACLR group^16,35^. On a neurophysiological level, we anticipate finding differences in the posterior and frontal ERSPs^12,16^, and in the task-related complexity of these regions^33^. Identifying kicking-specific deficits from multifaceted standpoints can motivate trainers and therapists to see an ACL injury not only as a peripheral injury, but also as a complex condition with neurophysiological aspects. This knowledge can elaborate diagnostics and interventions on a neurophysiological and task-specific basis and improve the longevity of athletic performance in football following injury.

## Results

### Descriptive data and task-related pain

The descriptive data of both groups are presented in Table 1. Analysis of demographic data and Marx Activity Scale (MAS) revealed no statistically significant differences between the ACLR group and healthy football players in terms of age (z = 1.63, *p* = 0.10), height (t^23^ = -1.08, p = 0.29), BMI (t^23^ = -0.15, p = 0.89), or activity level (t^23^ = -0.41, p = 0.69). Knee Injury and Osteoarthritis Outcome (KOOS) indicated the existence of knee symptoms within the ACLR group. Despite this, participants reported no pain during the kicking task as measured by Visual Analogue Scale (VAS).

**Table 1:** The participants’ demographic data and activity level. Self-reported knee function and task-related pain are presented for the ACLR group.

**Demographic data**	ACLR Group (n = 10)	Healthy controls (n = 15)	Mean differences (*p value*)
Age (years, med (IQR))	25.50 (3)	20.50 (4.50)	0.10
Sex (female / male)	4 / 6	6 / 9	-
Height (cm)	174.20 ± 10.42	177.80 ± 6.25	0.29
Body mass index (BMI, kg/m^2^)	23.20 ± 2.66	23.34 ± 2.09	0.89
Time post-surgery (months, med(IQR))	5.05 (3.60)	-	-
Graft type	Semitendinosus (n = 6) Quadriceps (n = 1) Patellar (n = 3)	-	-
**MAS**	14 ± 2.11	13.47 ± 3.74	0.69
**KOOS**			
Pain (%)	76.32 ± 8.46	-	-
Symptoms (%)	72.23 ± 10.52	-	-
Activities of daily living (%)	76.30 ± 15.34	-	-
Sport and recreation (%)	77.89 ± 18.07	-	-
Quality of life (%)	69.13 ± 5.51	-	-
**Task-related Pain (VAS)**	0	-	-

### Accuracy rate

The mean accuracy rates were 80.56 ± 11.22 for the ACLR group and 84.74 ± 8.60 for the healthy controls. No statistically significant difference was observed between the two groups (t^23^ = 1.05, *p* = 0.30).

### Trial-to-trial fluctuations in the kicking motion

**Fig. 1:**
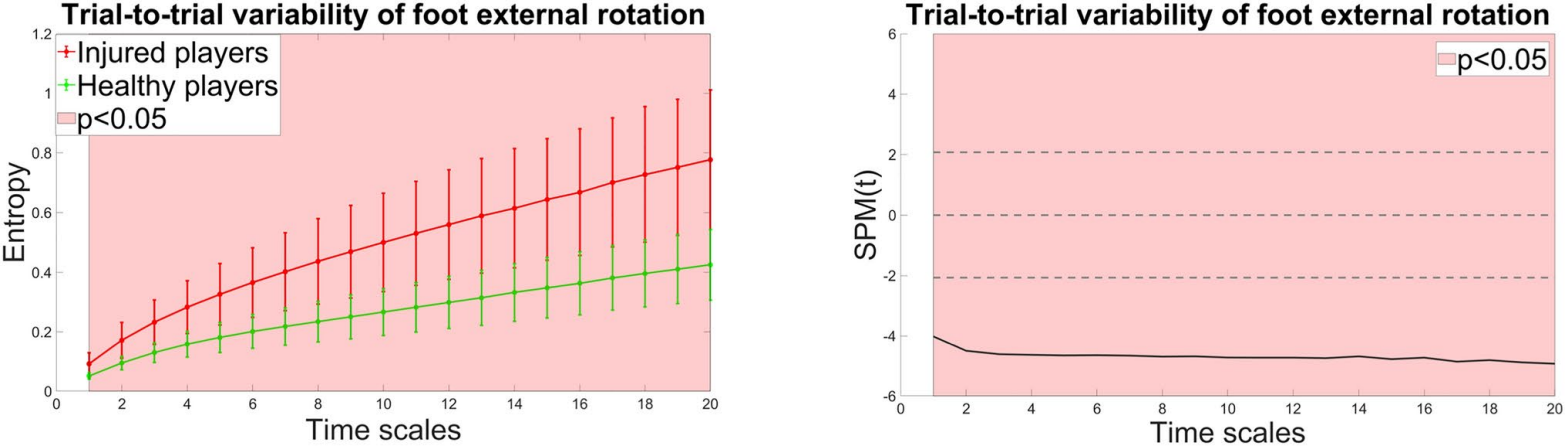
Entropy estimates of injured and healthy players computed for foot external rotation demonstrating statistically significant differences along 20 time scales (left) and the corresponding statistical parametric map (SPM, right). In SPM, the solid line displays computed *t*-values for each time scale, with critical *t*-values for both tails indicated by the dotted line. Areas where the computed *t*-value exceeds the critical threshold show time scales with statistically significant differences.

There were no statistically significant differences between groups in entropy estimates for hip flexion and knee flexion across all temporal scales. However, the ACLR group exhibited significantly higher entropy values for foot external rotation across all temporal scales (all p values < 0.005), with differences becoming more pronounced at coarser scales. The statistical parametric mapping is presented in Fig. 1.

No significant correlations were observed between pass accuracy and the entropy estimates of hip flexion, knee flexion and foot rotation across the analyzed temporal scales.

### Kick-related spectral perturbations

**Fig. 2:**
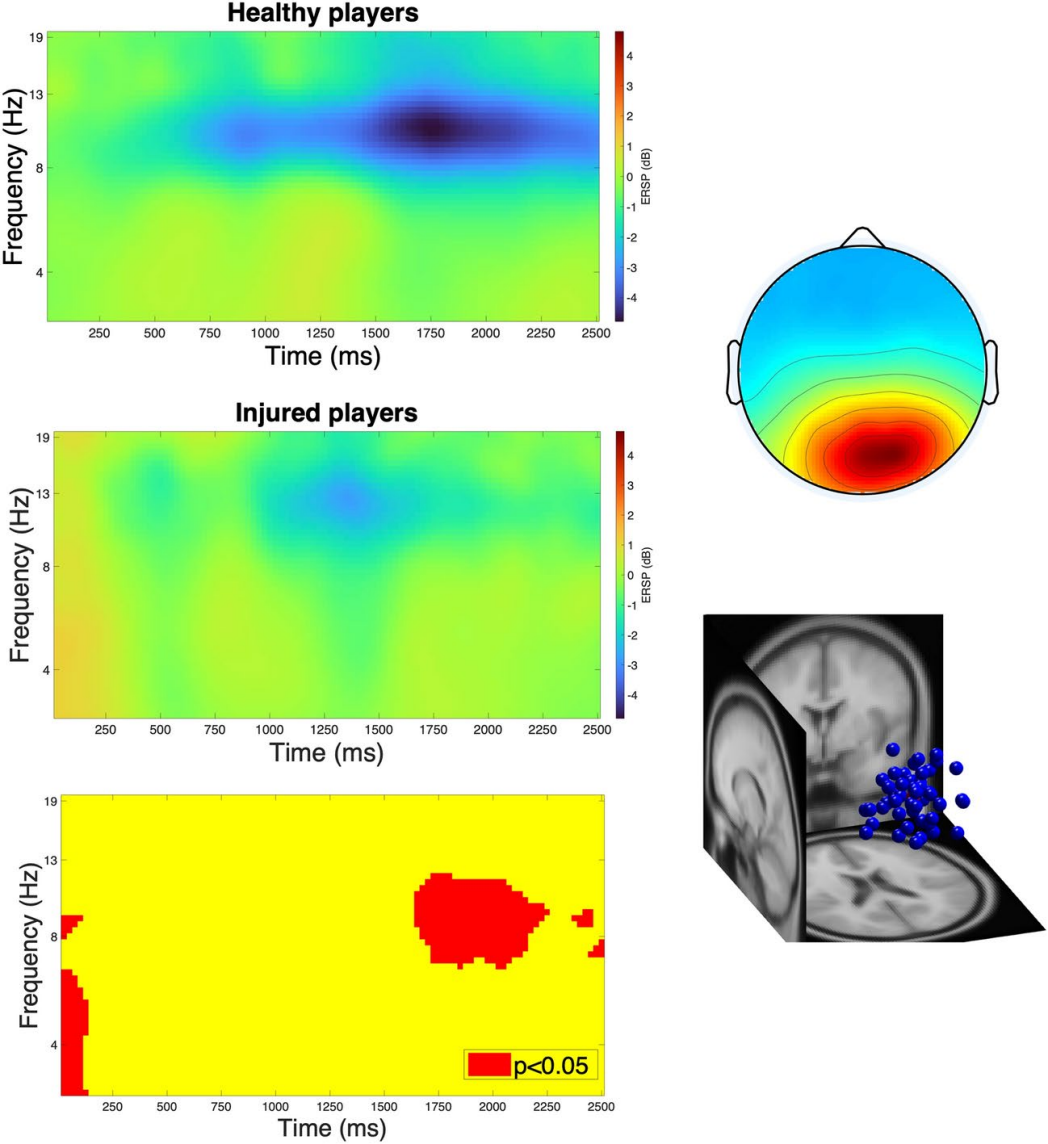
Kick-related spectral perturbations in the right posterior cluster showing a stronger alpha desynchronization in healthy players (left column, top) compared to injured players (left column middle) with significant differences at 1750—2250 ms (left column, bottom). “0” ms indicates kick onset. The right column demonstrates the scalp map of the cluster (top) and the locations of allocated independent components (bottom).

Independent components (IC) allocated to the right posterior cluster (N = 24 [n_healthy_ = 15, n_injured = 9_], N_IC_ = 49 [n_healthy_ = 33, n_injured_ = 16]) exhibited an alpha desynchronization following kick onset, which was more pronounced in healthy players. In contrast, a more pronounced theta synchronization was observed in the ACLR group at kick onset. These patterns resulted in statistically significant differences between the groups in the time windows of 1750—2500 and 0—125 ms (Fig. 2).

**Fig. 3:**
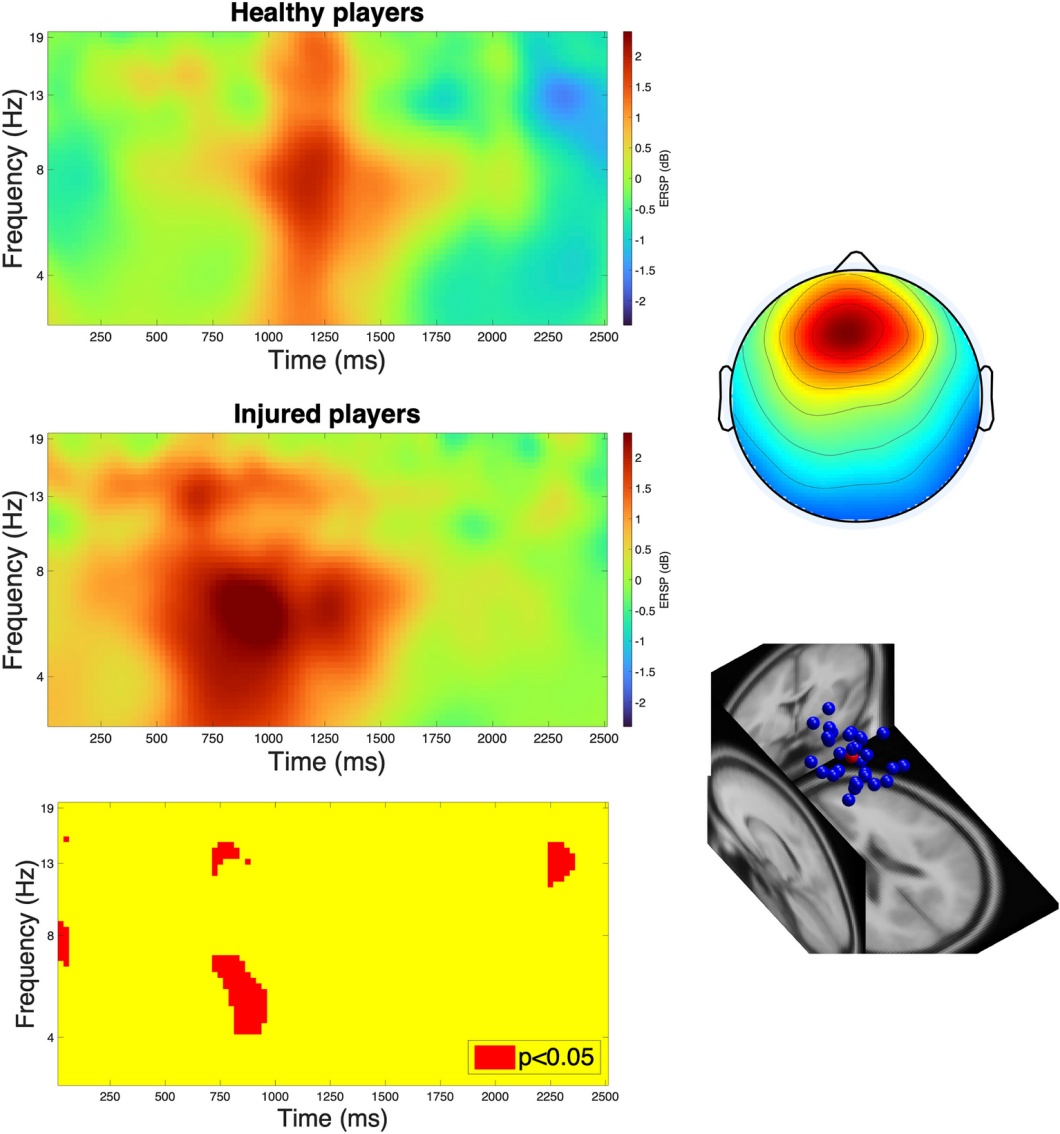
Kick-related spectral perturbations in the frontal cluster showing a more pronounced theta synchronization in injured players (left column, middle) compared to healthy players (left column, top) with significant differences at 750—1000 ms (left column, bottom). “0” ms indicates kick onset. The right column demonstrates the scalp map of the cluster (top) and the locations of allocated independent components (bottom).

For the mid-frontal ICs (N = 18 [n_healthy_ = 12, n_injured_ = 6], N_IC_ = 30 [n_healthy_ = 22, n_injured_ = 8]), a theta synchronization was noted, which was more intense in the ACLR group from the onset of the kick. Statistically significant differences between groups were observed in the time window of 750—1000 ms corresponding to this pattern (Fig. 3).

### Brain complexity related to kicking

**Fig. 4:**
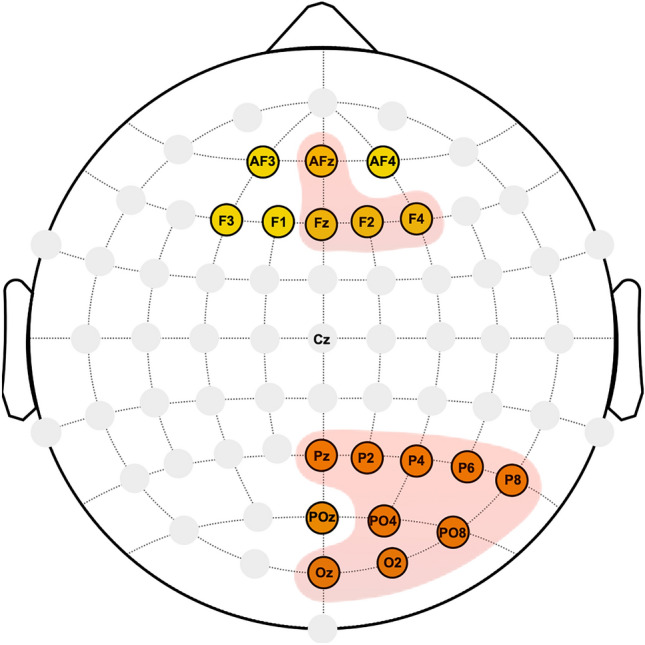
Channels indicating significant differences between healthy and injured players (shaded red) in the complexity of right posterior (orange-colored) and mid-frontal (yellow-colored) regions.

**Fig. 5:**
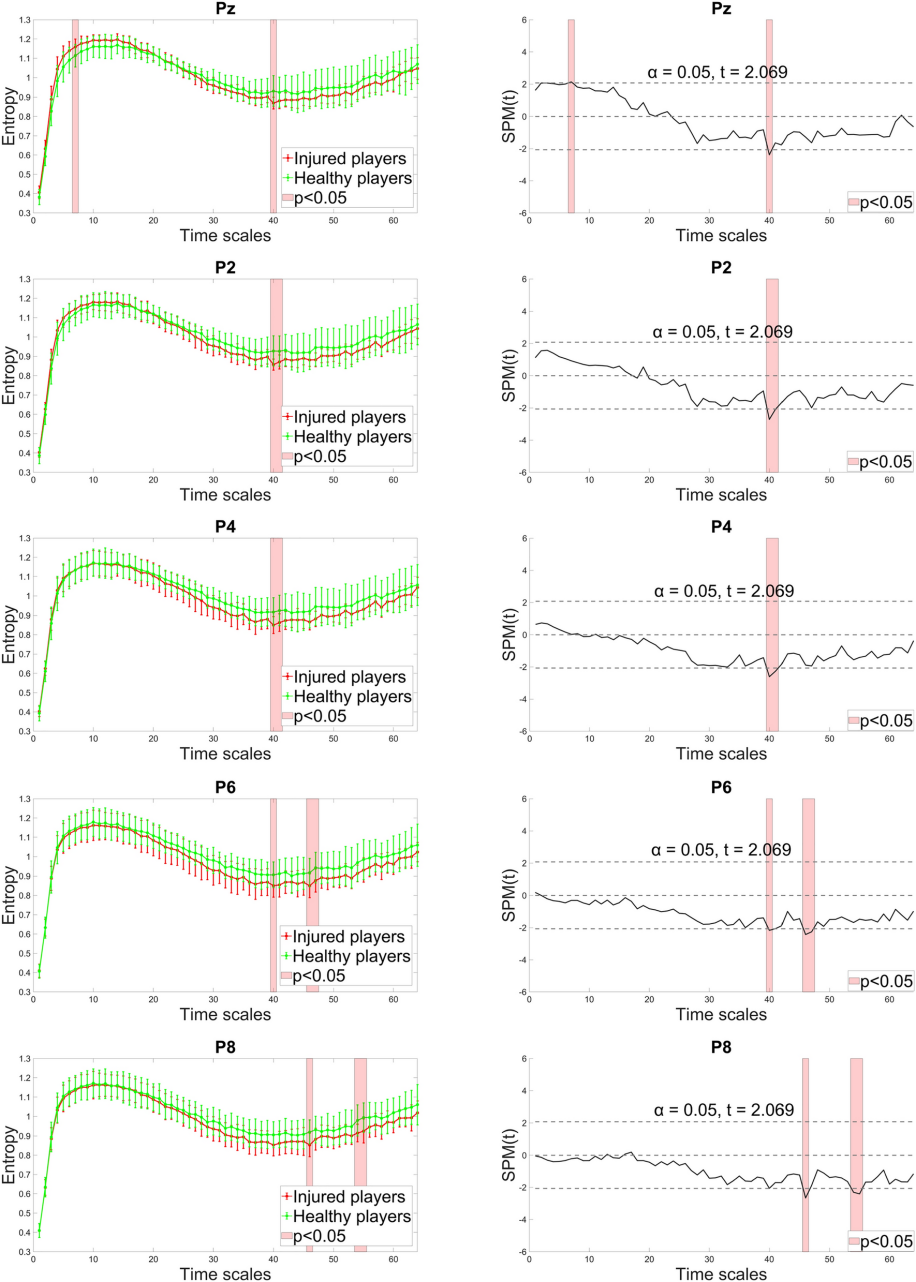
Entropy estimates of Pz, P2, P4, P6 and P8 along 64 time scales (left column) and the statistical parametric mapping (SPM) of significant differences observed between injured and healthy players (right column). Injured players demonstrated lower complexity at medium and coarse scales with significant differences observed in a range between 40 and 56. For Pz, the lower trend reversed to higher complexity at fine scales yielding significant differences at scale 7. In SPM, the solid line displays computed *t*-values for each time scale, with critical *t*-values for both tails indicated by the dotted line. Areas where the computed *t*-value exceeds the critical threshold show time scales with statistically significant differences.

**Fig. 6:**
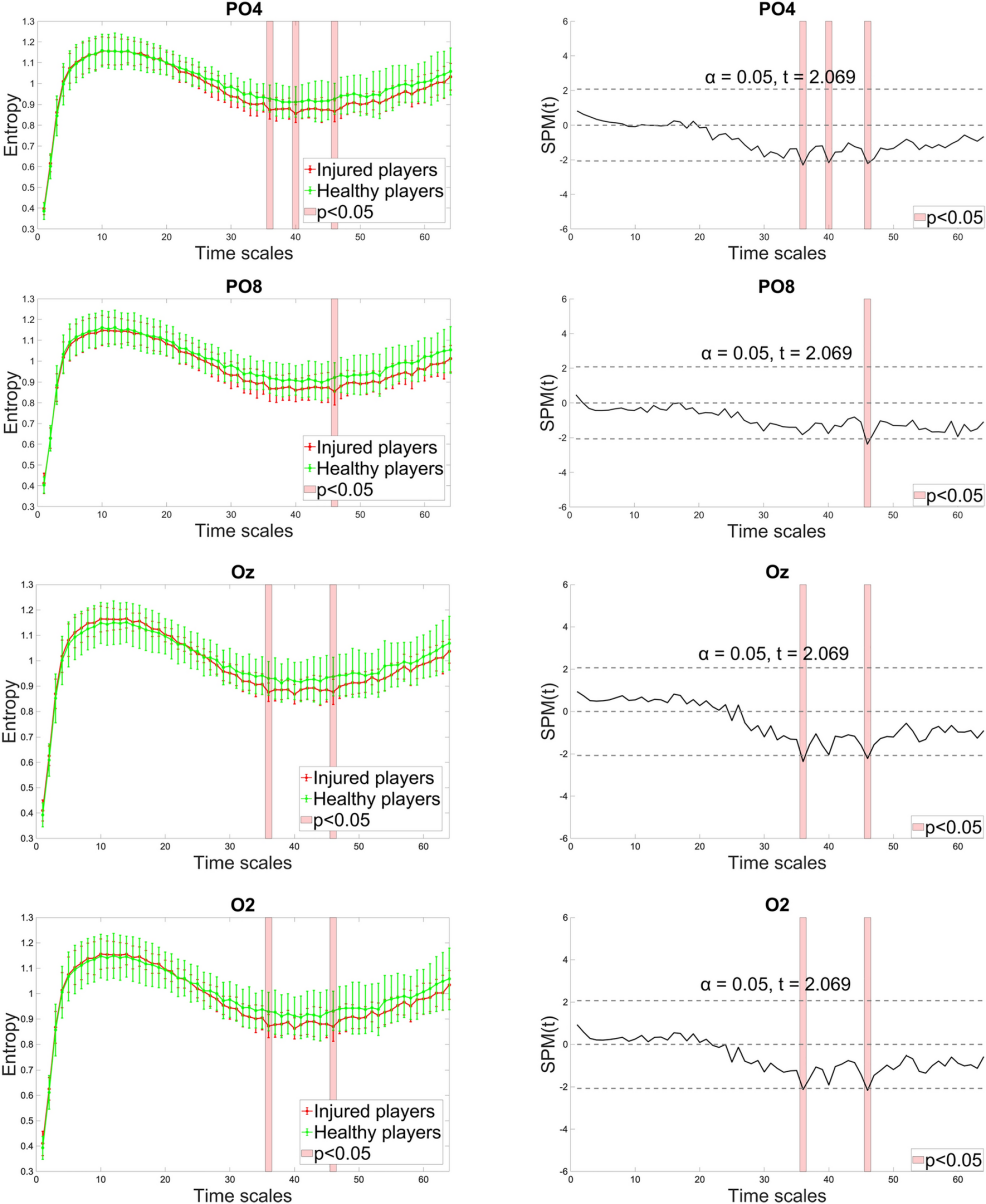
Entropy estimates of PO4, PO8, Oz and O2 along 64 time scales (left column) and the statistical parametric mapping (SPM) of significant differences observed between injured and healthy players (right column). Injured players demonstrated lower complexity at medium and coarse scales with significant differences observed in a range between 36 and 46. In SPM, the solid line displays computed *t*-values for each time scale, with critical *t*-values for both tails indicated by the dotted line. Areas where the computed *t*-value exceeds the critical threshold show time scales with statistically significant differences.

In the right posterior area, no significant differences were found between groups for the POz electrode (Fig. 4). However, in the other channels, the ACLR group demonstrated a tendency for lower entropy at medium and coarse scales, with occasional significance in the range of scales 36—56. For the Pz, this trend reversed, showing higher entropy at fine scales, with significant differences observed at scale 7. Scales with significant entropy differences and the corresponding statistical parametric maps are presented in Fig. 5 and 6.

**Fig. 7:**
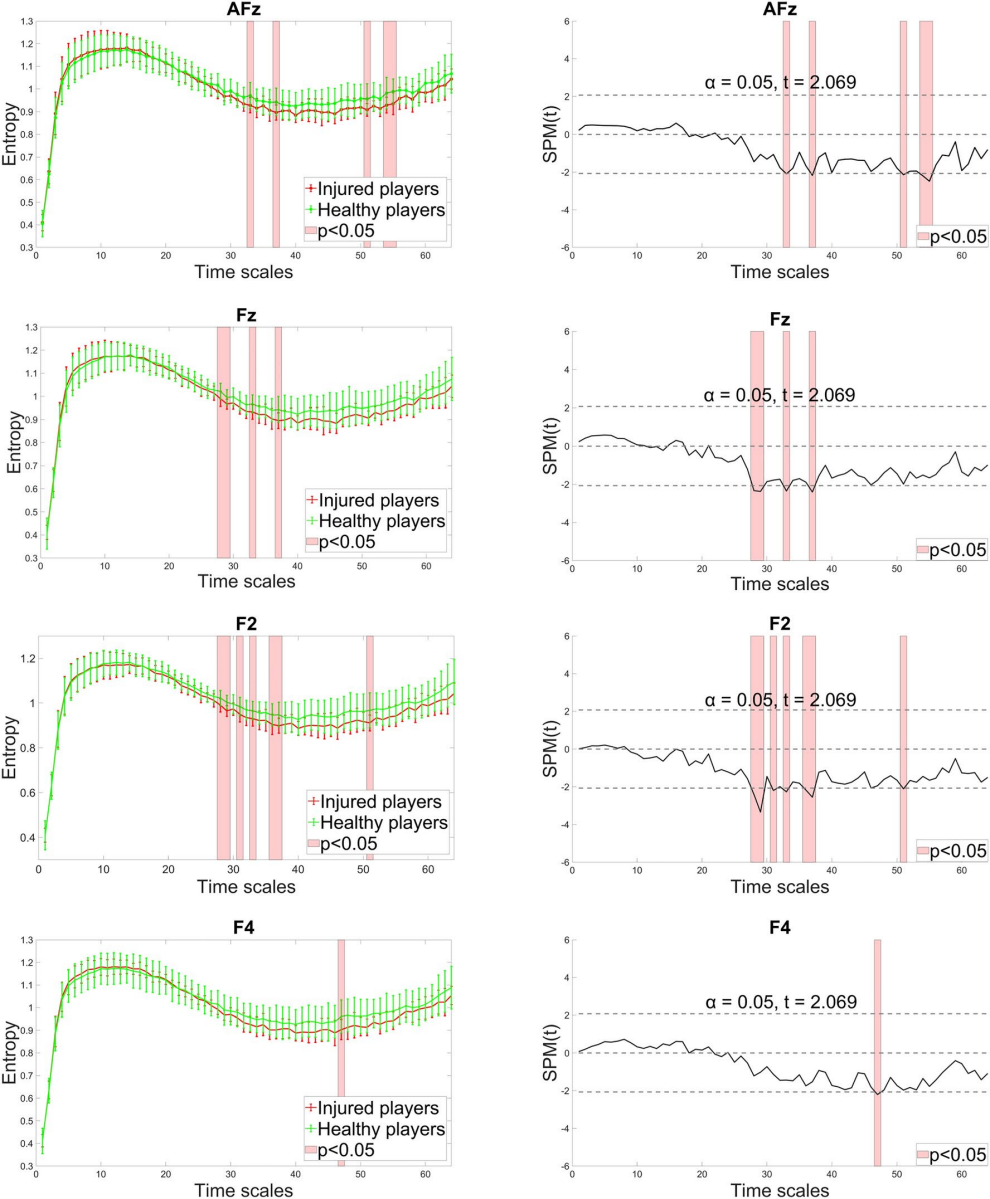
Entropy estimates of AFz, Fz, F2 and F4 along 64 time scales (left column) and the statistical parametric mapping (SPM) of significant differences observed between injured and healthy players (right column). Injured players demonstrated lower complexity at medium and coarse scales with significant differences observed in a range between 27 and 56. In SPM, the solid line displays computed *t*-values for each time scale, with critical *t*-values for both tails indicated by the dotted line. Areas where the computed *t*-value exceeds the critical threshold show time scales with statistically significant differences.

In the mid-frontal area, entropy estimates for the AF3, AF4, F3 and F1 electrodes did not differ significantly between groups (Fig. 4). However, significant differences were found for AFz, Fz, F2 and F4, predominantly at medium and coarse scales in the range of 27—56, with the ACLR group showing lower entropy estimates. Scales with significant entropy differences and the corresponding statistical parametric maps are presented in Fig. 7.

## Discussion

The current study compared kicking performance and the associated cortical activity in the posterior and frontal regions between healthy and football players who have undergone an ACLR. Despite the lower trend in the ACLR group, the accuracy was not significantly different between the groups. The trial-to-trial complexity of hip and knee flexion motions showed no significant differences. However, entropy estimates of foot external rotation indicated higher complexity in the ACLR with incremental differences toward coarser scales. Kick-related spectral perturbations revealed differences in cortical activity associated with kicking. ACLR revealed a weaker posterior alpha desynchronization and a stronger frontal theta synchronization upon kick onset. Furthermore, the complexity of these regions was lower at medium and coarse scales, with Pz exceptionally also at fine scales.

### Kicking performance

Although healthy and injured players demonstrated statistically comparable accuracy in kicking performance, the consistency of motor behavior associated with this performance, specifically the trial-to-trial complexity of foot external rotation, was significantly higher in the ACLR group. While linear measures of movement variability reduce variations observed between trials to randomness and hereby lose the temporal structure of a repetitive pattern, MSE quantifies the complexity of this variability as a non-linear measure by assaying temporal fluctuations that occur within multiple repetitions of a task across multiple time scales^36–38^. An optimal level of variability in a movement pattern is considered indicative of healthy motor function, while decreased or increased variability reflects rigid and noisy movement patterns, respectively, and can be detrimental to performance^38^. Consistent with other studies reporting a loss of optimal variability following injury^35^, our findings suggest a noisier movement for injured players with less consistency from one trial to another^39^.

The significant difference observed solely in the variability of foot external rotation can be attributed to the spatial demands inherent in executing a precise kick towards a target. From the perspective of the kicking leg, foot external rotation results from a combination of tibial torsion and hip external rotation in the transverse plane^40^. The alignment of the foot relative to the target is crucial for determining the ball’s trajectory while kicking^41^. Injured individuals present changes in knee rotational angles due to disrupted proprioceptive acuity^42^, which may lead to difficulties in dynamic control and execution of kicks with distorted angles^43^. Consequently, attempts to optimize spatial accuracy through varying rotational angles may increase variability in the transverse plane during the kicking motion. From the perspective of the support leg, the torque generated during the swing phase facilitates pelvic rotation, thereby promoting accurate external rotation of the kicking leg within the proximal-to-distal kinematic chain^44^. However, when proprioceptive acuity is impaired^42^, inaccuracies in pelvic rotation may indirectly affect the rotational angles of the kicking leg, leading to diminished spatial consistency^45^.

Analysis of complexity across different time scales revealed increasing group differences in foot external rotation particularly at coarser scales. In MSE, the coarse-graining procedure removes local details in a signal towards higher time scales and reveals global trends. This suggests higher unpredictability of the broader kicking pattern in injured players upon removal of rapid, local movement fluctuations^46^. In the context of injury, this could indicate a lack of stable, repeatable strategies or difficulties in achieving consistent coordination across trials due to deficiencies of higher-order adaptability^35,47^. Considering our previous findings that revealed higher complexity in novices compared to experienced players performing the same task^16^, it can be postulated that ACL injury may affect the ability to maintain a reinforced spatial kicking pattern.

The lack of significant differences in accuracy despite complexity differences, together with the absence of a correlation between the two, underscores the independence of outcome and motor processes. Injured players may maintain accuracy through compensatory strategies, with complexity serving as an indicator of altered motor control. Furthermore, this decoupling may also highlight task-dependency as low demands may mask underlying sensorimotor deficits. The relationship between variability and accuracy may emerge as task difficulty increases.

### Kick-related spectral perturbations

The comparison of spectral perturbations induced by kick onset in the right posterior and frontal regions revealed significant differences between healthy and injured football players. Several neuroimaging studies have demonstrated distinct activity patterns in these regions during the execution of simple motor tasks^17–19^, postural tasks^21,27,48^ and dynamic movements^28^ following an ACLR. Among EEG studies examining power spectrum measures, inconsistent findings regarding increased or decreased posterior alpha and frontal theta activity have been reported^17,18,27,28,48^, potentially due to the task-specific nature of injury-related compensatory strategies.

The posterior cluster was characterized by an alpha desynchronization during kicking. Alpha suppression is known to correlate directly with cortical activation levels^49,50^ and associated with visual processes in posterior regions^51–54^. Our previous studies utilizing the same task revealed a similar alpha response across comparably distributed spatial clusters, underscoring the visuospatial demands inherent in kicking^12,16^. The rightward laterality of the ICs in the current findings aligns with our earlier findings^12,16^ and may accent the maintenance of visual attention to a spatial location predominantly managed by the non-dominant hemisphere^55^. However, in the ACLR group, the alpha desynchronization was diminished with significant differences. Injured individuals are known to rely more on visual cues during movement^19–21,56^. This phenomenon of cross-modal compensation^57,58^ suggests that an injury may lead to increased visual processing, resulting in more pronounced posterior activity during motor tasks^19,48^. Conversely, our findings indicate reduced posterior activity in injured individuals, potentially suggesting an attenuated integration of visuospatial information.

The discrepancy of current and former findings may stem from the relatively lower visuospatial demands of previously implemented tasks such as knee flexion / extension or single-leg stance. In contrast, kicking necessitates interaction with the ball and target, employing concurrent visual and attentional processes^12,15,16^. In injured players, compensatory mechanisms for sensory deficits may restrict available cortical resources and lead to conflicts between compensation strategies and task demands^24,58^. Increased cognitive load through dual task paradigms has been shown to diminish performance in either cognitive or motor domains among injured individuals, implying a weighted allocation of cortical resources towards either task demands or compensatory efforts^24,59–62^. However, cognitive demands are inherently integrated into kicking as a prerequisite to perform effectively^63^, utilizing visual and attentional resources during execution to accurately perceive the target and fine-tune positioning^12,16^. Considering a potential overlap of cortical regions engaged in compensation and task execution, the diminished posterior activation may reflect challenges in maintaining allocated attention on visual targets. In addition to differences observed in alpha band activity, the stronger posterior theta synchronization noted in injured players shortly after kick onset may also be indicative of increased cortical load, as previous research has shown that theta power also tends to increase in posterior cortices when visual and physical demands become more challenging in dynamic tasks^27,64^.

Furthermore, the stronger frontal theta response observed in injured players supports the notion of an interplay between sensory compensation and task demands. Midline frontal theta is widely recognized as a marker of cognitive processes, particularly attentional engagement^65–67^. Previous studies have reported increased frontal theta activity among injured individuals during simple and dynamic tasks^17,18,28^, highlighting an augmented need for attention during movement. In the present study, while injured football players achieved comparable spatial accuracy to their healthy counterparts, they did so by drawing upon greater attentional resources, a known strategy employed to facilitate diminished sensorimotor integration^68,69^. Moreover, the distribution of mid-frontal ICs in our findings encompasses premotor cortices^70,71^, involved in motor planning and online movement correction^72,73^. Given that theta activity within these regions has been shown to increase alongside task demands^74,75^, this response may further suggest a necessity for more intensive planning processes in injured players. Integrating our findings on both posterior and frontal spectral dynamics, it can be speculated that football players prioritize attentional strategies for sensorimotor compensation during kicking. Consequently, this prioritization may constrain available resources necessary for addressing the visuospatial demands inherent in this complex motor task following ACLR.

### Task-related cortical complexity

In addition to differences in linear spectral dynamics, entropy estimates revealed distinct non-linear temporospatial characteristics in the right posterior and frontal cortical activity between healthy and injured football players while kicking. For both regions, the MSE curves indicate lower cortical complexity in injured players, particularly at medium and coarse scales^76^. Although no prior studies have investigated the neurophysiological consequences of musculoskeletal injuries through the lens of complexity theory, a loss of complexity has been frequently observed in various pathological conditions such as neurological and cardiac disorders^76–80^. Higher complexity is considered indicative of greater information-processing capacity^81,82^ and operates at a critical edge between randomness and regularity^83^. This critical balance is essential for adaptability^30,84^ and the capacity to transition swiftly between states of randomness and regularity, which is vital for optimal cognitive flexibility^82,85^. Hung et al.^86^ found that the superior performance of expert rifle shooters is associated with lower task-related cortical complexity. Their findings suggest that expertise in sports reduces neuromotor noise by optimizing the balance between randomness and regularity. In contrast, our findings suggest that in injured football players, the cortical complexity in the posterior and frontal regions may remain below this critical threshold during kicking, resulting in a reduced amount of information integrated into neural networks compared to their healthy counterparts^83^.

The reduced complexity observed in injured players is particularly notable at coarser scales. In MSE analysis of EEG signals, fine-to-coarse scales characterize the complexity of fast-to-slow neural dynamics^34^, which correspond to the integrated information into local-to-distributed networks, respectively^32^. This lower distributed entropy in injured football players may reflect their attenuated capacity to integrate information processed in right posterior and frontal regions into long-range networks during kicking. McIntosh et al.^32^ have discussed a similar shift towards local processing in older adults within a modularity framework^87^, proposing that a decrease in distributed entropy may indicate reduced interdependence among spatially distant brain regions. Although the mechanisms underlying this shift remain covert, given that complexity findings are limited to only two regions of interest, it is plausible that proprioceptive compensation in injured players might reduce the resources available for global integration (decreased modularity), which is accepted as an important catalyzer for a complex, adaptive behavior^88–90^. Given its proximity to the sensorimotor cortex, the higher complexity observed at fine scales in the Pz region may imply a more intensive sensorimotor processing in injured players, hypothetically interfering with the long-range integration of information. However, the lack of comparable entropy findings related to movement limits these interpretations to preliminary assumptions. Nevertheless, the observed injury-related loss of complexity in these two regions could be linked to decreased movement consistency, as these areas are crucial for superior execution^15,16^.

The current study presents preliminary evidence suggesting that the neurophysiological consequences of an ACL injury, investigated within postural and static motor tasks up to date, may extend to kicking, a fundamental movement in a sport significantly affected by this injury^1–3^. Our results indicate that the allocation of visual and attentional strategies differ in injured players during kicking compared to their healthy counterparts, potentially impacting the integration of task-related information in posterior and frontal regions. Achieving comparable spatial accuracy to their healthy counterparts even in a stable, predictable environment may compel injured players to compensate for deficits with augmented attention, possibly at the cost of restricted visual resources necessary for effective kicking performance^15,16^. The resultant decrease in cortical complexity and increase in trial-to-trial movement complexity may signal a regression in athletic skills^16,86,91,92^ and potentially presage reduced performance in real-world scenarios with more unpredictable dynamics. Our study provides the first insight into how ACL injury might affect kicking performance in football from a neurophysiological perspective and highlights the potential for further research in the field of sports-related injuries.

### Methodological considerations and prospect

The current study has several methodological limitations that should be considered when interpreting the findings, translating them into practice, and designing future studies that build on this work. Regarding the characteristics of the injured cohort, while neurophysiological changes have been reported years after ACL rupture^19,93,94^, the present findings may primarily reflect distinctions observed during the return-to-sports phase, which could develop chronically over time^95^. However, the absence of pre- and post-injury comparisons introduces uncertainty about injury-specific nature of the findings^96,97^. Pre-existing hip abnormalities, which have been identified as potential contributors to ACL injury^98,99^, may offer an alternative explanation for the increased variability in external rotation observed in this study.

The described dynamics suggest the existence of behavioral and cortical differences between healthy and injured football players while kicking. However, the heterogeneity arising from the concurrence of ACLR in kicking- and stance-leg, complicates the clear identification of side-specific deficits. Given the differing demands of the legs during a kick (for instance, proprioceptive acuity and stability^44,45^, injuries to the kicking and stance leg may result in distinct cortical strategies detectable particularly in motor cortices. Furthermore, although injured individuals did not report any perceived pain during the execution of the task, prolonged pain exposure is known to influence cortical activity^100,101^. Considering KOOS outcomes, this should be acknowledged as a potential confounding factor. Future studies should also consider the homogeneity of autografts, as outcomes may vary among different types^102,103^.

The kicking task used in this study was adopted from our previous research^12,16^ and is intended to simulate short-distance passes on the field, where accuracy prevails^10,104^. However, the predictability of the experimental environment may limit the ecological validity of the task and could also explain the insignificant or small differences observed. Increasing attentional demands in future studies could exacerbate kicking-specific deficits, making them more apparent. Consequently, future studies should design settings with greater unpredictability and difficulty to better reflect real-world conditions, such as adaptive environments, variable motor tasks and unpredictable sensory cues^105^.

## Conclusions

The current study aimed to investigate the potential impact of ACL injury on kicking performance from a neurophysiological standpoint. Despite similar accuracy performance, injured football players demonstrated notable behavioral and cortical differences compared to their healthy counterparts during kicking. The higher entropy observed in foot external rotation may suggest a loss of optimal variability, likely due to diminished proprioceptive acuity. This reduction in proprioceptive function may be compensated for by increased attentional demands, which could limit the use of visuospatial resources and hinder the integration of task-related information into long-range neural networks. Our preliminary findings offer the first insights into how the established neurophysiological consequences of ACL injury might affect kicking performance in football and open doors for prospective research to further explore this topic, given its practical and academic potential.

## Methods

### Participants

The current study investigated 10 football players who had undergone ACLR (n_right_ = 6) and 15 healthy controls. The inclusion criteria for the injured players were as follows: (1) aged between 18—35 years; (2) having undergone ACLR due to a complete rupture of the ACL, with or without accompanying meniscal injury; (3) having played football for at least 10 years prior to injury^16^; (4) experiencing no pain during the execution of the kicking task; (5) being right-dominant in lower extremities; and (6) not having any previous ACL or concomitant injuries, neurologic disorders or being on psychotropic medication. For the healthy control group, the inclusion criteria included: (1) aged between 18—35 years; (2) playing football for a minimum of 10 years^16^; (3) being right-dominant in the lower extremities; (4) having no orthopedic injuries, neurological disorders, or being on psychotropic medication. The activity levels of both groups were assessed and compared using MAS^106^. Lower extremity laterality was determined based on the Lateral Preference Inventory^107^. KOOS was utilized to evaluate self-reported knee function among injured players prior to the experiments^108^. Given its influence on cortical activity^100,101^, task-related pain levels were assessed using a VAS. All participants had normal or corrected vision during the experiments. The Ethics Committee of Paderborn University approved the study’s conduction and written consent was obtained from all participants prior to measurements. All methods were performed in accordance with the relevant guidelines and regulations.

### Experimental procedure

The current study employed a short-distance kicking task, which has been used in our previous research (see Supplementary Fig. 1 online)^12,16^. All participants wore laced sneakers during the experiments and performed the task in a standardized area within the laboratory. The objective was to kick a FIFA size five ball with the inside of the right foot toward a target, represented by a rectangular wooden block (10 × 15 cm). To ensure consistency across trials, the starting position was standardized. Participants placed their left foot next to the ball while positioning their right foot externally rotated behind it. The distance between the left foot and the ball was established during familiarization trials and marked on the floor to maintain this standardized position throughout the experiment. Participants were instructed to kick as accurately as possible, and no interactions occurred during trials. A total of six blocks, each consisting of 15 repetitions, were completed. This setup has previously demonstrated reliable cortical dynamics in our findings^12^. During the experiments, the number of successful (on-target) kicks and missed attempts were recorded to calculate the accuracy rate.

### The recording and multiscale entropy analysis of behavioral data

Seven wearable inertial measurement unit sensors (myoMOTION, Noraxon, USA) were utilized to capture the three-dimensional biomechanics of the lower extremities at a sampling rate of 200 Hz. These sensors were bilaterally attached to the dorsal surfaces of the feet, the tibial faces of the shanks, the lateral lower quadrants of the upper thighs, and the sacral surface of the lumbar area^109^. The recorded signals for hip flexion, knee flexion, foot external rotation, and acceleration were digitized using myoRESEARCH (version 3.14, Noraxon, USA) and processed for complexity analysis in Matlab (version R2020b, The Math Works, USA).

As a non-linear measure, MSE assesses the complexity of temporal variations in a time series by identifying repeating patterns across multiple temporal resolutions^36,38^. In a time series where each repeating pattern corresponds to fluctuations within an individual trial, MSE evaluates the regularity of these patterns, and hereby the regularity of trials. Lower entropy values indicate high predictability and consistent patterns, while higher entropy values reflect irregularity or inconsistency from trial to trial. The coarse-graining procedure removes local trends by smoothing the series at larger time scales and facilitates the understanding of trial-to-trial changes also in global movement^46^. Based on these assumptions, the current study assessed kicking consistency across the trials using MSE. The continuous data were processed into sparse segments based on kick onsets to remove intervals unrelated to kicks and to normalize drifts caused by variations in the starting position^16^. Kick onsets were identified by minimizing a cost function over possible linear change points in the x-axis of the acceleration data^44,110^. The signal was rectified and smoothed using a Gaussian-weighted moving average filter with a window length of 200 data points^111^. Abrupt changes in the mean acceleration signal were detected as kick onsets using *ischange* function in Matlab. The signals for hip flexion, knee flexion, and foot rotation were segmented into intervals of 500 data points (2500 ms) based on kick onsets with each segment representing a trial^16^. The segments were then concatenated and the baseline of the resulting signal was shifted to a constant value of zero^112^ to increase the stationarity of the data without altering its structure and eliminate the confounding effect of starting position variations across trials on entropy estimates^113^. Spline interpolation was applied to transition points to avoid artificial fluctuations caused by abrupt changes and smooth the adjunction. To avoid the manipulation of small fluctuations, data was not filtered^113,114^. The processing steps are presented with an example dataset in Supplementary Fig. 2 online.

Custom scripts were used to perform MSE analysis in two steps predicated on methodologies established by Costa et al.^36^. Firstly, time series were derived across 20 time scales through a coarse-graining procedure defined as:where *T* represents the time scale, *y*_j_ is the constructed time series after coarse-graining, *x*_i_ is a data point within that series, and *N* denotes the length of the original time series. Next, sample entropy for each generated time series was estimated as:

with the parameters *m* = embedding dimension, *N* = the total number of data points, *r* = similarity criterion and *n* = the number of vectors close to a template vector *i* with a dimension of *m.* An embedding dimension of *m* = 2 was selected, with the similarity criterion defined as 0.50 × SD of the time series. The selection of *m* should consider the structure of the fluctuations and its functional meaning in the series, as patterns at a length of *m* and *m* + 1 are searched in the algorithm^113^. In the current study, *m* = 2 was selected to capture motion fluctuations characterized by this length at fine and coarse scales (see Supplementary Fig. 2 online). For the highest scale analyzed, a minimum length range of 14^ m^ – 23^ m^ (196 – 529 for *m* = 2) are recommended^115^, which was fulfilled for all datasets at scale 20. In the traditional MSE algorithm, *r* remains constant across time scales; however, this can inflate entropy values for shorter time scales due to reduced SD following coarse-graining procedures^116^. To address this issue in the current study, we recalculated *r* for each time scale^117^.

### EEG recording and preprocessing

Cortical activity was continuously recorded using 65 active Ag/AgCl electrodes (actiCap, Brain Products, Germany) positioned according to the international 10–20 system, with AFz and FCz serving as the ground and reference electrodes, respectively^118^. The signal was transmitted and digitized at a sampling rate of 500 Hz using BrainVision Recorder (Brain Products, Germany) and a wireless amplifier (LiveAmp, Brain Products, Germany). Additionally, a 3D accelerometer (Brain Products. Germany) was affixed posterior to the lateral malleolus to capture the acceleration of the kicking synchronously.

Data preprocessing was performed offline in Matlab (version R2020b, The Math Works, USA) using the EEGLAB toolbox (version 14.1.2b)^119^. Sinusoidal line noise was removed via the Cleanline plugin^120^, followed by filtering the signal with a basic finite impulse response filter with cut-off frequencies set at 3 and 30 Hz. Automatic detection and rejection of noisy channels were conducted based on kurtosis, probability, and signal spectrum, with a *z*-score threshold = 5^121^. Subsequently, the data were re-referenced to a common average and downsampled to 256 Hz.

The preprocessed data were then segmented into epochs based on kick onset detection, which was performed using principles of linear computational cost^110^. The acceleration data of the kicking foot along the x-axis was rectified and smoothed using a Gaussian-weighted moving average filter with a window length of 1000 data points. Kick onsets were identified as abrupt changes in the signal mean using the *ischange* function, and the data were epoched from -3000 to 3000 ms, with baseline correction applied from -2500 to -500 ms^12,16^. Noisy epochs were visually inspected and removed from the data.

An adaptive mixture independent component analysis was performed to further analyze the data, decomposing the epoched data into maximally independent components (IC)^122^. Source localization of ICs was estimated using the DIPFIT plugin^123^ with a standardized four-shell spherical head model (BESA, Germany). Robust brain components were retained based on their source location, residual variance (< 15%) and classification as brain components by the ICLabel Plugin^124^, with a minimum 90% as confidence threshold. All non-brain ICs were subsequently removed from the dataset.

### Computation of event-related spectral perturbations in the clusters of interest

The retained brain components were clustered using the *k*-means algorithm. To avoid circular inference in subsequent statistical analyses, dipole locations were used to construct the global distance matrix^125^. The optimal number of clusters was determined through the application of three distinct optimization algorithms^126–128^. Guided by our previous research^12,16^, the right posterior and frontal clusters were identified as clusters of interest for further analysis.

ERSP matrices for these two clusters were computed in line with the methodology outlined by Makeig^129^. The analysis focused on a frequency range of 3 to 20 Hz and a time window extending from 0 to 2500 ms^12,16^, utilizing the integrated STD_ERSP function in the EEGLAB toolbox. A 3-cycle wavelet was employed for the lowest frequency, with a linear increase of 0.5 cycles as the frequency rose.

### Multiscale entropy analysis in the regions of interest

A modified MSE analysis was performed on the preprocessed EEG data using a custom algorithm, based on the methodology established by Grandy et al.^130^. MSE analysis of EEG data requires tailored preprocessing protocols, given that factors like sampling rate, band-pass filter parameters, and artifact rejection techniques can significantly impact entropy estimates^131,132^. In mobile EEG studies, movement artifacts are of particular concern requiring special preprocessing considerations^133,134^. Due to the absence of standardized preprocessing pipelines for MSE analysis in mobile settings, the current study adopted procedures from our previous work, producing reliable results for ERSPs^12^.

Channels removed during preprocessing were subsequently interpolated^135^. To ensure consistency across participants, 80 epochs—the minimum number of clean epochs retained among datasets following the rejection of noisy epochs—were randomly selected using the POP_SELECT function. This step was critical to compute entropy estimates with an equal number of channels and data points per channel, ensuring reliable results^130^. The selected epochs were concatenated, and entropy estimates were calculated for 64 scales (with the equations provided in the above section) on the residuals of the EEG signal, following subtraction of the intra-individual average across trials. This approach is known to yield reliable outcomes for discontinuous data^130,132^.

The analysis was conducted over the 0—2500 ms epoch window to focus on the complexity of task-related activity as outlined in Piskin et al.^12^. An embedding dimension of *m* = 2 was used, with the similarity criterion recalculated for each scale as *r* = 0.50 × SD, to avoid bias toward lower entropy values at coarser scales^131,132^. Two regions of interest were defined based on our previous findings^12,16^ to assess the complexity in the mid-frontal (AF3, AFz, AF4, F3, F1, Fz, F2, F4) and right posterior areas (Pz, P2, P4, P6, P8, POz, PO4, PO8, Oz, O2). Finally, entropy values were extracted from the specified channels for statistical analysis.

### Statistical analyses

An a priori sample-size estimation was performed using G*Power^136^, based on the behavioral findings by Cordeiro et al.^25^. The observed difference in knee extension variability during kicking with a large effect size (*d* = 1.215) indicated that a minimum number of 11 in the healthy and 9 participants in the ACLR group would be required to achieve a power of 0.80 at an alpha level of 0.05.

Statistical analyses were performed using Matlab (version R2020b, The Math Works, USA). The normality of descriptive data was assessed using the Shapiro–Wilk-Test^137^. Variables following a normal distribution were reported as mean ± SD and were compared using an independent *t*-test. For skewed data, median and interquartile ranges (IQR) were presented, and group comparisons were made using the Wilcoxon rank-sum test.

The ERSP matrices were compared on a pixel-wise basis using integrated permutation-based *t*-tests in EEGLAB, with false discovery rate correction to map *p*-values and identify group differences with temporal and frequency specificity^16^. Entropy estimates of the behavioral and EEG data were compared across scales using independent *t*-tests^138^, with statistical parametric maps (SPM) generated to display significant group differences at fine (1—21), medium (22—42), and coarse (42—64) scales^139^. A false discovery rate correction was applied following the Benjamini and Hochberg method^140^ to account for simultaneous statistical inferences. In order to examine the influence of trial-to-trial complexity on kicking accuracy, Pearson’s correlation coefficients were computed for accuracy rate and entropy estimates across analyzed time scales. All analyses were conducted with a significance level set at 0.05.

## Supplementary Information

Supplementary Information 1.

Supplementary Information 2.

## Data Availability

The datasets collected and analyzed for the present work are available from the corresponding author on reasonable request.
