# Supplementary material for: Cortical changes associated with an anterior cruciate ligament injury may retrograde skilled kicking in football: preliminary EEG findings
Source: Sci Rep. 2025 Jan 16;15:2208. doi: 10.1038/s41598-025-86196-4 (PMC11739489; doi:10.1038/s41598-025-86196-4)
Supplement: Supplementary file 1 — Supplementary Information 1. [file 41598_2025_86196_MOESM1_ESM.pdf]

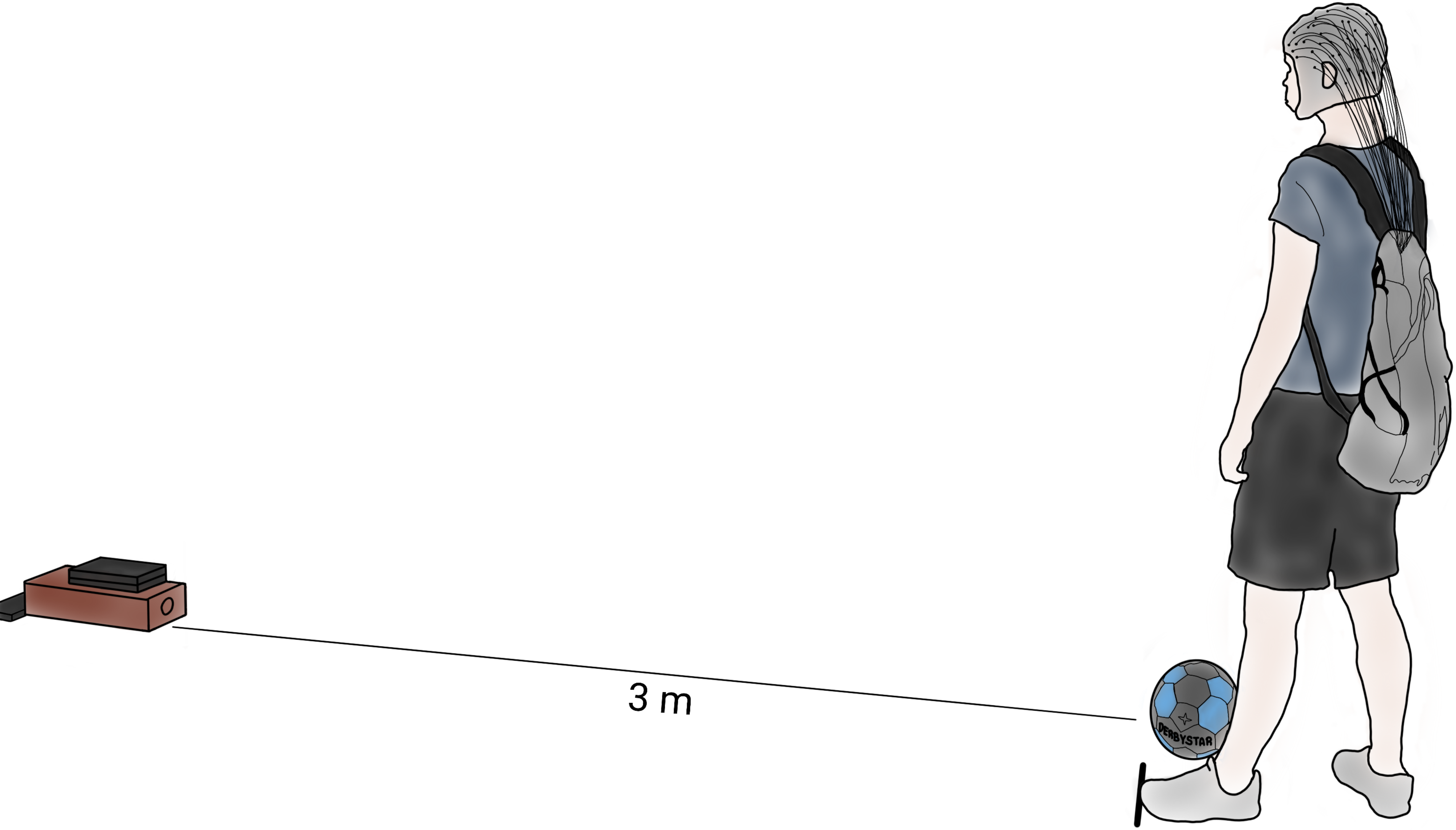

The short-distance kicking task. Participants executed instep kicks with their dominant (right) foot towards a target placed at 3 meters.
