# Supplementary material for: Cortical changes associated with an anterior cruciate ligament injury may retrograde skilled kicking in football: preliminary EEG findings
Source: Sci Rep. 2025 Jan 16;15:2208. doi: 10.1038/s41598-025-86196-4 (PMC11739489; doi:10.1038/s41598-025-86196-4)
Supplement: Supplementary file 2 — Supplementary Information 2. [file 41598_2025_86196_MOESM2_ESM.pdf]

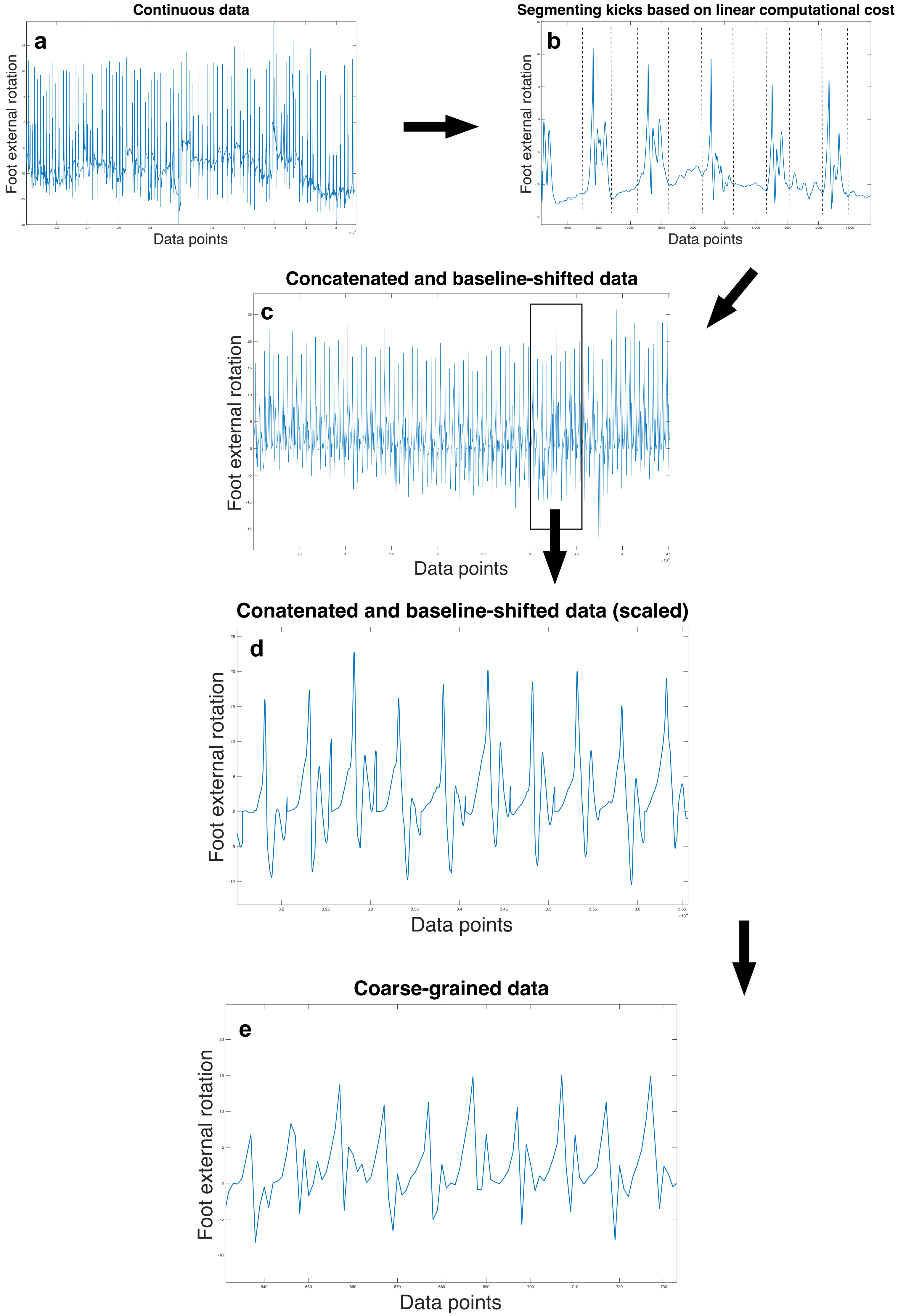
Continuous data
a
Foot external rotation
Data points
Segmenting kicks based on linear computational cost
b
Foot external rotation
Data points
Concatenated and baseline-shifted data
c
Foot external rotation
Data points
Conatenated and baseline-shifted data (scaled)
d
Foot external rotation
Data points
Coarse-grained data
e
Foot external rotation
Data points
